# Somatostatin and CXCR4 chemokine receptor expression in hepatocellular and cholangiocellular carcinomas: tumor capillaries as promising targets

**DOI:** 10.1186/s12885-017-3911-3

**Published:** 2017-12-28

**Authors:** Daniel Kaemmerer, Robin Schindler, Franziska Mußbach, Uta Dahmen, Annelore Altendorf-Hofmann, Olaf Dirsch, Jörg Sänger, Stefan Schulz, Amelie Lupp

**Affiliations:** 1 Department of General and Visceral Surgery, Zentralklinik Bad Berka, Bad Berka, Germany; 2 Institute of Pharmacology and Toxicology, Jena University Hospital, Friedrich Schiller University Jena, Drackendorfer Str. 1, D-07747 Jena, Germany; 3 Department of General, Visceral and Vascular Surgery, Jena University Hospital, Jena, Germany; 4 Institute of Pathology, Jena University Hospital, Jena, Germany; 5 Institute of Pathology and Cytology Bad Berka, Bad Berka, Germany

**Keywords:** Somatostatin receptors, Chemokine receptor, CXCR4, Hepatocellular carcinoma, Cholangiocellular carcinoma

## Abstract

**Background:**

Hepatocellular (HCC) and cholangiocellular carcinomas (CCC) display an exceptionally poor prognosis. Especially for advanced disease no efficient standard therapy is currently available. Recently, somatostatin analogs have been evaluated for the treatment of HCC, however, with contradictory results. Besides, for both malignancies the chemokine receptor CXCR4 has been discussed as a possible new target structure.

**Methods:**

Expression of somatostatin receptor (SSTR) subtypes 1, 2A, 3, 4, and 5, and of CXCR4 was evaluated in a total of 71 HCCs and 27 CCCs by immunohistochemistry using well-characterized novel monoclonal antibodies.

**Results:**

In HCC tumor cells, frequency and intensity of expression of SSTRs and CXCR4 were only low. CXCR4 was present in about 40% of the HCCs, although at a low intensity. SSTR5, SSTR2, and SSTR3 were detected in about 15%, 8%, and 5% of the HCC tumors, respectively. SSTR and CXCR4 expression was much higher in CCC than in HCC. CXCR4 and SSTR1 were present in 60% and 67% of the CCC samples, respectively, followed by SSTR2 and SSTR5, which were detected in 30% and 11% of the tumors, respectively. Most notably, CXCR4 was intensely expressed on the tumor capillaries in about 50% of the HCCs and CCCs. CXCR4 expression on tumor vessels was associated with poor patient outcomes.

**Conclusions:**

CCC, but not HCC, may be suitable for SSTR-based treatments. Because of the predominant expression of SSTR1, pan-somatostatin analogs should be preferred. In both HCC and CCC, indirect targeting of tumors via the CXCR4-positive tumor capillaries may represent a promising additional therapeutic strategy.

**Electronic supplementary material:**

The online version of this article (10.1186/s12885-017-3911-3) contains supplementary material, which is available to authorized users.

## Background

Hepatocellular carcinomas (HCCs) account for 90% of primary liver tumors and are the fifth most common malignoma diagnosed in men, the seventh most common malignoma diagnosed in women, and the third leading cause of cancer-related death worldwide. The highest incidence is reported for China, Africa, and South-East Asia (>20 cases per 100,000 persons), whereas HCCs are relatively uncommon in Western countries (<5 cases per 100,000 persons) [1, 2]. On the whole, HCC displays a very poor prognosis, with an overall median survival of 11 months and an overall 1-year survival rate of less than 50% [3, 4]. Especially for advanced disease, there is no efficient standard therapy currently available. The only curative treatment options are liver resection and liver transplantation, providing 5-year survival rates of up to 70%. However, only a minority of patients (<20%) is suited for such interventions because of tumor expansion, metastatic disease, severe cirrhosis, and impaired liver function [1, 5]. Further treatment modalities, especially for advanced-stage disease, comprise transarterial chemoembolization (TACE), ethanol injection, radio-frequency or microwave ablation, and selective internal radiation therapy (SIRT). With respect to systemic pharmacotherapy, which currently consists only of sorafenib, a multi-tyrosine kinase inhibitor with antiangiogenic and antiproliferative properties, a significant therapeutic benefit has been shown in patients with advanced HCC, whereas “classical” chemotherapy is ineffective in such patients [1, 5]. However, sorafenib rapidly loses efficacy in many cases [6, 7], because of additional mutations in the signal transduction cascades. Therefore, alternate (tyrosine) kinase inhibitors and combined therapy regimens, in addition to other targeted therapies, are currently under investigation for the treatment of advanced HCC [6].

Intrahepatic cholangiocarcinomas (CCCs) are the second most common type of primary liver tumor and account for about 10% of all primary malignancies of the liver [1, 8]. The prognosis for CCC is exceptionally poor, such as the best outcomes, seen in patients who are candidates for surgery, show a 5-year survival rate of 30.6% and a median overall survival of 27 months following diagnosis [9]. Because of high recurrence rates, liver transplantation is not recommended in CCC. Liver resection represents the only curative treatment option; however, CCC is diagnosed relatively late in the majority of patients, making curative resection no longer possible [1]. Compared with the best supportive care, systemic chemotherapy with gemcitabine and cisplatin significantly prolongs survival of patients with inoperable tumors, making it the treatment standard for advanced disease, although overall benefits are not very pronounced. Therefore, other targeted therapies are urgently needed also for CCC [10, 11].

Among the new molecular target structures that have been evaluated for treatment of HCC are the somatostatin receptor (SSTR) subtypes SSTR1 – SSTR5, which in immunohistochemical studies have been shown to be present in a substantial fraction of tumor samples, although quite divergent expression intensities and receptor expression profiles have been described [12–17]. Since data obtained from in vitro experiments using hepatoma cell lines and from different HCC animal models were also very promising [18–24], a number of clinical investigations was conducted to investigate the prognostic and therapeutic value of SSTRs. Apart from a few promising studies [16, 25–28], most trial outcomes were quite discouraging (see e.g. [29–36]). The reasons for these discrepancies are still unknown and remain to be elucidated.

The chemokine receptor CXCR4 represents another promising molecular target structure for tumor diagnosis and therapy. Overexpression of this receptor has been observed in more than 20 different tumor entities and many studies demonstrated that increased CXCR4 expression is associated with rapid tumor progression, high invasiveness, early metastasis, and poor patient outcomes [37]. With respect to HCC, however, the data are contradictory. In the majority of studies, CXCR4 expression was higher in tumors than in normal liver tissues and was correlated with tumor aggressiveness and reduced patient survival [38–44]. Other immunohistochemical or PCR analyses demonstrated either no difference [45, 46] or even lower CXCR4 expression in tumors [47, 48] compared with normal tissues. Therefore, further investigations in the role of CXCR4 in HCC are necessary.

In contrast to HCC, CCCs have not been evaluated for SSTR expression so far and only one study has examined CXCR4 expression in CCC [49], demonstrating correlations between CXCR4 expression and tumor progression, metastasis, and poor patient outcomes.

The vast majority of the existing immunocytochemical and immunohistochemical data on SSTR and CXCR4 expression in HCC and CCC was obtained using polyclonal antibodies from different commercial and non-commercial sources, which may explain the divergent results obtained. Additionally, in many cases (especially with respect to CXCR4) even a nuclear staining for these membrane-bound G-protein-coupled receptors has been described, suggesting non-specific antibody interactions. Therefore, the aim of the present study was to re-evaluate the expression of SSTRs and CXCR4 in a large set of formalin-fixed, paraffin-embedded HCC and CCC samples by using well-characterized novel rabbit monoclonal antibodies. These antibodies were generated and extensively characterized by our group [50–54] and have also been validated by other authors (e.g. [55, 56]). In parallel and to re-evaluate SSTR and CXCR4 expression in hepatoma cell lines, samples of three commonly used human hepatoblastoma (HepG2) and HCC cell lines (Hep3B, and HuH-7) as well as of the small cell lung cancer cell line NCI-H69 and the pancreatic neuroendocrine tumor cell line BON-1 (as positive controls) were embedded in paraffin blocks and stained for the different SSTRs and CXCR4.

## Methods

### Tumor specimens

A total of 171 archived, formalin-fixed, paraffin-embedded tumor samples from 98 patients (in detail, 78 × 1, 2 × 2, 7 × 3, 3 × 4, 1 × 5, 2 × 6, 3 × 7, 1 × 8, and 1 × 10 samples per patient) with histologically verified HCC or CCC were included in the present investigation. Of the 98 tumors investigated, 71 (72%) were HCC and 27 (28%) were CCC. Samples were provided by the Institute of Pathology and Cytology Bad Berka, Bad Berka, Germany, and by the Institute of Pathology, Jena University Hospital, Jena, Germany. The tumors had been surgically removed between 2007 and 2014 either at the Department of General and Visceral Surgery, Zentralklinik Bad Berka, Bad Berka, Germany, or at the Department of General, Visceral and Vascular Surgery, Jena University Hospital, Jena, Germany. The corresponding clinical data were gathered from patient records.

### Cytoblocks

HepG2, Hep3B, HuH-7, NCI-H-69, and BON-1 cells (DSMZ, Braunschweig, Germany) were grown in 75 cm^2^ culture flasks to a confluency of 80%. Cells were washed once with phosphate-buffered saline and transferred into 10% buffered formalin (J.T.Baker, Deventer, The Netherlands) for 2 h. After centrifugation for 10 min at 3500 x *g*, the supernatant was removed, and 1 ml human pool plasma was added to the cell samples. After brief vortexing, 100 μl human fibrinogen (50–70% protein; ≥80% clottable; Sigma-Aldrich Chemie GmbH, Steinheim, Germany) was added to each sample, and the samples were vortexed again. The resulting clots were placed for another 24 h in 10% buffered formalin and embedded in paraffin blocks.

### Immunohistochemistry

From the paraffin blocks, 4 μm sections were cut, floated onto adhesive-coated slides and subjected to immunostaining by means of an indirect peroxidase labeling method as described recently [57]. Except for SSTR4, rabbit monoclonal antibodies (hybridoma cell culture supernatants) were used to detect SSTRs and CXCR4 (Table 1; [57]). As positive controls, sections from human pancreas (islets; SSTR1, SSTR2, SSTR3, SSTR5), lymph nodes (germinal centers; SSTR2, SSTR5, CXCR4, Ki-67), and human cortex (SSTR4) were used (Additional file 1: Figure S1). As negative control, the primary antibody was either omitted or adsorbed for 2 h at room temperature with 10 μg/ml of the peptide used for immunizations of the rabbits [57]. Additional immunostainings were performed with monoclonal mouse antibodies against the proliferation marker Ki-67 (Table 1).

**Table 1:** Antibodies used for immunohistochemical stainings

Antibody	Clone	Type	Epitope	Supplier	Dilution
SSTR1	UMB-7	rabbit monoclonal	ENLESGGVFRNGTCTSRITTL (residues 377–391)	Epitomics, Burlingame, CA	1:25
SSTR2	UMB-1	rabbit monoclonal	ETQRTLLNGDLQTSI (residues 335–369)	Epitomics, Burlingame, CA	1:10
SSTR3	UMB-5	rabbit monoclonal	QLLPQEASTGEKSSTMRISYL (residues 398–418)	Epitomics, Burlingame, CA	1:20
SSTR4	4802	rabbit polyclonal	CQQEALQPEPGRKRIPLTRTTIF (residues 366–388)	Gramsch, Schwabhausen, Germany	0.1 μg/ml
SSTR5	UMB-4	rabbit monoclonal	QEATPPAHRAAANGLMQTSKL (residues 344–364)	Epitomics, Burlingame, CA	1:10
CXCR4	UMB-2	rabbit monoclonal	KGKRGGHSSVSTESESSSFHSS (residues 338–359)	Epitomics, Burlingame, CA	1:2
Ki-67	MIB-1	mouse monoclonal		DAKO, Hamburg, Germany	1:75

Staining for the receptors was evaluated by means of the semiquantitative Immunoreactivity Score (IRS) according to Remmele and Stegner [58] and as described recently [57]: The percentage of positively stained tumor cells quantified in five gradations (no positive cells [0], <10% positive cells [1], 10–50% positive cells [2], 51–80% positive cells [3], >80% positive cells [4]) was multiplied by the staining intensity, which was assessed in four scales (no staining [0], mild staining [1], moderate staining [2], strong staining [3]). Finally, IRS values extending from 0 to 12 were obtained. In case of more than one tumor slide per patient, an arithmetic mean was calculated from the IRS values of these slides. Only tumor samples with IRS values ≥3 were considered positive [57]. IRS values were further classified as follows: 0–2, negative/no expression; 3–5, low expression; 6–8, moderately strong expression; 9–12, strong expression. Staining of the tumor vessels for SSTRs and CXCR4 was evaluated separately by determining the percentage of positive vessels in relation to all vessels. Tumors with ≥10% of vessels being stained for the respective receptor were considered positive [57]. With respect to Ki-67 staining, the percentage of positive nuclei was determined. All immunohistochemical stainings were evaluated by two independent blinded investigators (RS, AL). In case of discrepant scores, final decision was achieved by consensus.

### Statistics

For graphical data processing and statistical analysis, the IBM SPSS statistics program version 22.0.0.0 was used.

Expression levels (IRS values) of the SSTRs and of the CXCR4 in the HCC and CCC samples were presented as box plots, depicting the median value, the upper and lower quartile, minimum and maximum values, and outliers. The outliers were defined as follows: mild outliers (marked by circles): data that fall between 1.5 and 3 times above the upper quartile or below the lower quartile, respectively; extreme outliers (marked by asterisks): data that fall more than 3 times above the upper quartile or below the lower quartile, respectively. Since these outliers could not be attributed to technical errors (some stains were repeated for control), but may reflect the expression profile of a yet undefined subset of patients, they were not excluded from the statistical analyses.

Because the data were not normally distributed (Kolmogorov-Smirnov test), for the statistical analyses Mann-Whitney test, Kendall’s τ-b test, Chi-Square test, and Spearman’s rank correlation were performed. For survival analysis, the Kaplan-Meier method with a log-rank test was used. *P* values ≤0.05 were considered statistically significant. For the correlation calculations with the clinical data, correction for multiple comparisons was performed by using the Bonferroni-Holm procedure.

## Results

### Patient characteristics

Overall, tumors from 80 male (82% of the cases) and 18 female patients (18% of the cases) were evaluated in the present investigation. Of the 71 HCC patients, 57 were male (80% of the cases) and 14 female (20% of the cases), of the 27 CCC patients 23 were male (85% of the cases) and 4 were female (15% of the cases) (Table 2). Mean age of the HCC patients at diagnosis was 63.9 years (median: 65 years; range: 25–87 years) and that of the CCC patients 65.5 years (median: 66 years; range: 45–79 years) (Table 2). There was no significant difference between HCC and CCC patients with respect to patient age and gender.

**Table 2:** Patient characteristics. HCC: hepatocellular carcinoma; CCC: cholangiocellular carcinoma

	HCC	CCC	All tumors
Total no.	71	27	98
Sex			
male	57	23	80
female	14	4	18
Age (years)			
mean	63.9	65.5	64.4
median	65.0	66.0	65
Survival (months)			
mean	20.3	14.7	18.8
median	18.0	11.0	15.0
Stage (n)			
I	24 [34%]	8 [30%]	32 [33%]
II	20 [28%]	13 [48%]	33 [34%]
IIIA	18 [26%]	1 [4%]	19 [19%]
IIIB	0 [0%]	0 [0%]	0 [0%]
IIIC	1 [1%]	0 [0%]	1 [1%]
IVA	0 [0%]	3 [11%]	3 [3%]
IVB	8 [11%]	2 [7%]	10 [10%]
Grading (n)			
G1	13 [18%]	1 [4%]	14 [14%]
G2	43 [61%]	18 [66%]	61 [62%]
G3	15 [21%]	7 [26%]	22 [23%]
G4	0 [0%]	1 [4%]	1 [1%]

The median survival was 18 months for HCC patients (minimum: 0 months; maximum: 73 months), and 11 months for CCC patients (minimum: 2 months; maximum: 56 months) (Table 2). Twenty-four (34%) of the HCC patients and 8 (30%) of the CCC patients had stage I disease, 20 (28%) of the HCC patients and 13 (48%) of the CCC patients had stage II disease, 18 (26%) of the HCC patients and 1 (4%) of the CCC patients had stage IIIA disease, 1 (1%) of the HCC patients had had stage IIIB disease (CCC: 0%), 3 (11%) of the CCC patients had stage IVA disease (HCC: 0%), and 8 (11%) of the HCC patients and 2 (7%) of the CCC patients had stage IVB disease (Table 2). With respect to histological grading, 13 (18%) of the HCC and 1 (4%) of the CCC were grade 1, 43 (61%) of the HCC and 18 (66%) of the CCC were grade 2, 15 (21%) of the HCC and 7 (26%) of the CCC were grade 3, and 1 (4%) of the CCC were grade 4 (HCC: 0%) (Table 2). Of the 71 HCC patients 54 (76%) had liver cirrhosis. Of these patients with liver cirrhosis only 2 patients each had a history of hepatitis B viral infection or of an autoimmune hepatitis. From 8 patients the etiology of liver cirrhosis was not known/not reported in the patient files. All other patients suffered from alcohol-related liver disease. None of the CCC patients had liver cirrhosis and there was also no history of hepatitis B or C, autoimmune or alcoholic liver disease reported with these patients.

### Somatostatin and CXCR4 chemokine receptor expression in tumor samples

**Fig. 1:**
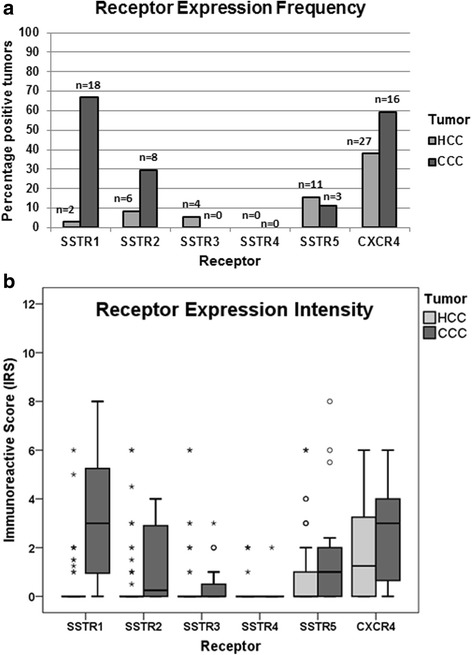
Expression profile of the different somatostatin receptor (SSTR) subtypes and of the CXCR4 chemokine receptor in hepatocellular (HCC) and cholangiocellular (CCC) carcinomas. **a** Percentage and number of positive cases for the different SSTRs and for CXCR4. Tumors were only considered positive at IRS values ≥3. **b** Box plots of the expression levels (IRS values) of SSTRs and CXCR4 in HCC and in CCC. Depicted are median values, upper and lower quartiles, minimum and maximum values, as well as outliers. Outliers are defined as follows: circles: mild outliers; data that fall between 1.5 and 3 times above the upper quartile or below the lower quartile; asterisks: extreme outliers; data that fall more than 3 times above the upper quartile or below the lower quartile

Only few of the HCC tumors displayed SSTR-positive or CXCR4-positive staining (i.e. an IRS value ≥3). Also median intensities of SSTR and CXCR4 expression (calculated from all IRS values ranging from 0 to 12) in the HCCs were only low (Fig. 1a and b; Table 3). CXCR4, the most prominent receptor in the HCC samples, was present in about 40% of the cases, but only at a very low intensity (median IRS: 1.25). Only four tumor samples (6% of the cases) displayed moderately strong CXCR4 expression with an IRS value ≥6. SSTR5, SSTR2, SSTR3, and SSTR1 were detected with IRS values ≥3 in about 15%, 8%, 6%, and 3% of the HCC tumors, respectively. No SSTR4 positivity was observed (Table 3). Median expression intensity for all SSTRs in the HCC samples amounted to an IRS value of 0 (Table 3). Eight (10%) of the patients displayed moderately strong (IRS value ≥6) SSTR expression in their HCC samples (SSTR1, 1 patient; SSTR2, 2 patients; SSTR3, 2 patients; SSTR5, 3 patients; no overlapping staining).

**Table 3:** Receptor expression data

	HCC	CCC
Total no.	71	27
mean IRS	0.41	3.50
median IRS	0	3.0
minimum/maximum IRS	0/6	0/8
positive cases (n)^a^	2 [3%]	18 [67%]
SSTR2		
mean	0.53	1.16
median	0	0.25
minimum/maximum IRS	0/6	0/4
positive cases (n)^a^	6 [8%]	8 [30%]
SSTR3		
mean	0.39	0.48
median	0	0
minium/maximum IRS	0/6	0/3
positive cases (n)^a^	4 [6%]	0 [0%]
SSTR4		
mean	0.15	0.07
median	0	0
minium/maximum IRS	0/2	0/2
positive cases (n)^a^	0 [0%]	0 [0%]
SSTR5		
mean	0.81	1.52
median	0	1.00
minium/maximum IRS	0/6	0/8
positive cases (n)^a^	11 [15%]	3 [11%]
cases with microvessel positivity (n)^b^	24 [34%]	12 [44%]
CXCR4		
mean	1.76	2.70
median	1.25	3.00
minium/maximum IRS	0/6	0/6
positive cases (n)^a^	27 [38%]	16 [60%]
cases with microvessel positivity (n)^b^	35 [50%]	15 [56%]

*HCC* hepatocellular carcinoma, *CCC* cholangiocellular carcinoma, *IRS* Immunoreactive Score; ^a^positivity for a receptor was adopted at an IRS value ≥3; ^b^tumors with ≥10% of the vessels being stained for the respective receptor were considered positive

Compared with the HCC samples, a larger fraction of the CCC samples displayed SSTR or CXCR4 staining (Fig. 1a and b; Table 3). CXCR4 was present with IRS values ≥3 in 60% of the CCC samples. SSTR1 was detected in 67% of the CCC samples. In both cases, the median IRS value was 3, which is, however, still low. SSTR2 and SSTR5 were expressed in 30% and 11% of the CCC samples, respectively. None of the CCC samples showed staining for SSTR3 or SSTR4. The median IRS value for SSTR5 was 1, whereas scores for SSTR2, SSTR3 and SSTR4 were below that level (Table 3). A total of eight CCC samples (30%) displayed moderately strong (IRS value ≥6) SSTR expression (7 samples for SSTR1, 3 samples for SSTR5). In two of the SSTR-positive tumors, both SSTR1 and SSTR5 were moderately co-expressed. Three (11%) of the CCC samples displayed moderately strong CXCR4 expression.

**Fig. 2:**
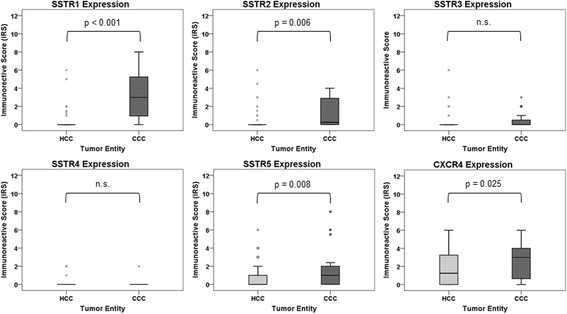
Differential somatostatin receptor (SSTR) and CXCR4 expression in hepatocellular and cholangiocellular carcinomas. Box plots of the expression levels (IRS values) of SSTRs and CXCR4 in hepatocellular and cholangiocellular carcinomas. Depicted are median values, upper and lower quartiles, minimum and maximum values, as well as outliers. Outliers are defined as follows: circles: mild outliers; data that fall between 1.5 and 3 times above the upper quartile or below the lower quartile; asterisks: extreme outliers; data that fall more than 3 times above the upper quartile or below the lower quartile

Comparing relative receptor expression intensities between the two tumor entities, CCCs showed significantly higher intensities of expression of SSTR1, SSTR2, SSTR5, and CXCR4 than HCCs (Fig. 2).

Regarding the most common receptor expression profiles (threshold for positive staining at IRS values ≥3), most of the HCC samples were negative for all of the receptors (*n* = 33; 46% of the cases). Nineteen of the HCC samples stained positive only for CXCR4, and four of the HCCs displayed double-staining for both SSTR5 and CXCR4. Nine of the HCC samples were positive for a single SSTR: three each for SSTR2, SSTR3, and SSTR5. Most of the CCC samples (*n* = 9; 33% of the cases) stained positive for both SSTR1 and CXCR4. Six of the CCC samples were negative for all of the receptors. Three of the CCC samples were positive for both SSTR1 and SSTR2.

In HCC, there was a significant correlation between the expression intensity of SSTR1 and the IRS values of SSTR4, SSTR5, and CXCR4 (Table 4). In CCC, there was a significant association between SSTR3 and SSTR5 expression intensities (Table 5). Additionally, there was a significant interrelationship between the proliferation marker Ki-67 and SSTR3 or SSTR5 expression intensities in HCC (SSTR3, *r* = 0.267, *p* = 0.024; SSTR5, *r* = 0.284, *p* = 0.016) and SSTR2 or SSTR5 expression intensities in CCC (SSTR2, *r* = 0.389, *p* = 0.045; SSTR5, *r* = 0.390, *p* = 0.044).

**Table 4:** Correlation between SSTR and CXCR4 expression intensities in hepatocellular carcinoma

	SSTR2	SSTR3	SSTR4	SSTR5	CXCR4
SSTR1					
r	0.101	−0.007	***0.258***	***0.235***	***0.236***
p	0.400	0.953	***0.030***	***0.048***	***0.048***
SSTR2					
r		0.079	0.117	0.162	0.043
p		0.511	0.331	0.178	0.724
SSTR3					
r			0.041	0.124	0.008
p			0.734	0.302	0.945
SSTR4					
r				0.178	0.066
p				0.138	0.584
SSTR5					
r					0.109
p					0.368

r: correlation coefficient (Spearman); p: *p* value; significant correlations (*p* ≤ 0.05) are marked in bold

**Table 5:** Correlation between SSTR and CXCR4 expression intensities in cholangiocellular carcinoma

	SSTR2	SSTR3	SSTR4	SSTR5	CXCR4
SSTR1					
r	0.380	0.052	−0.279	0.139	0.311
p	0.051	0.798	0.159	0.491	0.114
SSTR2					
r		0.277	0.241	0.290	−0.014
p		0.161	0.226	0.142	0.944
SSTR3					
r			0.344	***0.557***	0.242
p			0.079	***0.003***	0.223
SSTR4					
r				0.195	0.140
p				0.330	0.487
SSTR5					
r					0.142
p					0.480

r: correlation coefficient (Spearman); p: *p* value; significant correlations (*p *≤ 0.05) are marked in bold

**Fig. 3:**
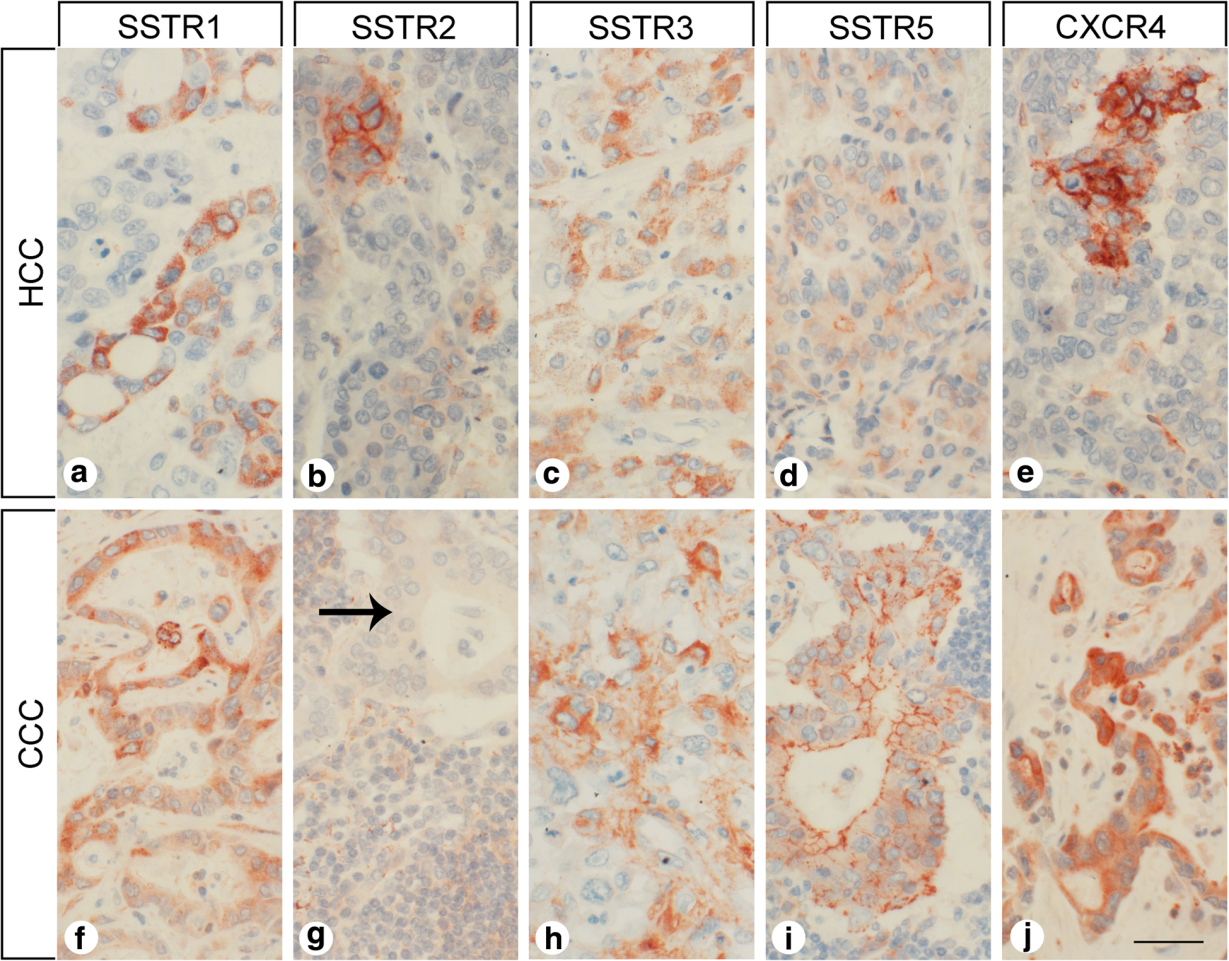
Somatostatin receptor (SSTR) and CXCR4 expression pattern in hepatocellular and cholangiocellular carcinomas. Depicted are typical examples of staining patterns for SSTR1 (**a, f**), SSTR2 (**b, g**), SSTR3 (**c, h**), SSTR5 (**d, i**), and CXCR4 (**e, j**). Immunohistochemistry (red-brown color), counterstaining with hematoxylin; scale bar: 50 μm; arrow in G: tumor cells. With the only exception of SSTR4, all receptors displayed both membranous and cytoplasmic expression

**Fig. 4:**
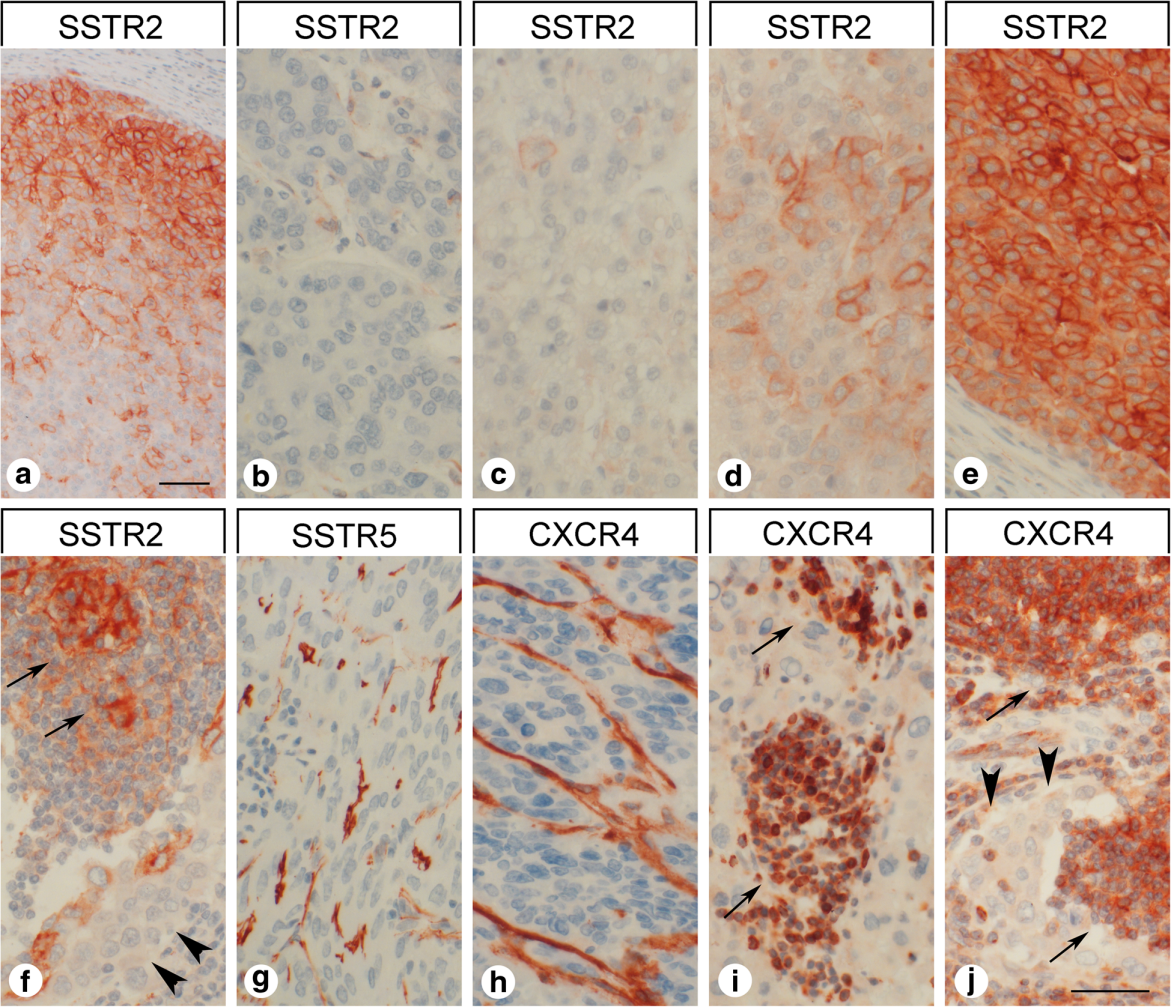
Intraindividual variability in somatostatin receptor 2 (SSTR2) expression intensity in hepatocellular and cholangiocellular carcinomas and presence of somatostatin receptors (SSTR) and of CXCR4 in surrounding normal tissue and in tumor stroma. **a-e** Typical example for intraindividual variability of SSTR2 expression intensity in hepatocellular carcinomas. All photomicrographs were taken from the same tumor slide. Immunohistochemistry (red-brown color), counterstaining with hematoxylin; scale bar: A: 60 μm, B-E: 50 μm. **f**, **j** Presence of SSTR2 and of CXCR4 in germinal centers of lymph follicles (arrows) in close vicinity to (largely negative) tumor cells (arrowheads). **g**, **h** SSTR5 and CXCR4 expression in tumor vessels. **i** Strong CXCR4 staining of infiltrating lymphocytes (arrows). Immunohistochemistry (red-brown color), counterstaining with hematoxylin; scale bar: 50 μm

As shown in Fig. 3, the monoclonal antibodies against SSTR1, SSTR2, SSTR3, SSTR5, and CXCR4 produced distinct immunostaining of the plasma membrane, but also of the cytoplasm of the HCC and CCC tumor cells. With the polyclonal anti-SSTR4 antibody, in contrast, only cytoplasmic immunostaining was observed (not shown). There was marked variation in staining intensity for all receptors within and among individuals, as depicted for SSTR2 in Figs. 4a–e, which show different photomicrographs taken from the same tumor slide. All in all, despite a few strongly stained tumor areas or groups of tumor cells, the tumors were largely negative in most cases, leading to low IRS values for each slide.

Independent from the tumor cells, many samples displayed strong staining for SSTR5 and CXCR4 on capillaries of the tumor stroma (Figs. 4g and h). SSTR5 expression on tumor microvessels was observed in 34% of the HCC samples and in 44% of the CCC samples. CXCR4 staining of the tumor microvessels was even detected in 50% of the HCC samples and in 56% of the CCC samples (Table 3).

Strong CXCR4 positivity was also seen in tumor-infiltrating lymphocytes (Fig. 4i). In the metastasis-containing lymph node samples, SSTR2 and CXCR4 were strongly expressed in germinal centers of (activated) lymph follicles in close proximity to the tumor cells (Fig. 4f and j). The tumor-surrounding “normal” liver tissues were largely devoid of any SSTR or CXCR4 expression. Occasionally, SSTR5 staining of bile duct epithelia or CXCR4 positivity of Kupffer cells was observed.

### Correlation with clinical data

In HCC a negative correlation between tumor diameter and SSTR2 expression intensity of the tumor cells was observed (r_sp_ = −0.319, *p* = 0.024). In CCC, on the other hand, tumor diameter was positively associated with intensity of CXCR4 expression of the tumor cells (r_sp_ = 0.762, *p* < 0.001). Here, CXCR4 positive tumors displayed a significantly larger diameter as compared to CXCR4 negative ones (Mann-Whitney test: *p* = 0.018). Apart from that, in both HCC and CCC, no significant correlation was seen between SSTR or CXCR4 expression intensities or positivity of the tumor cells and TNM classification, staging or grading of the tumors, presence or absence of cirrhosis or of vascular infiltration, presence of a solitary tumor or of multiple lesions, height of ASAT, ALAT or GGT serum values or of the tumor markers alpha fetoprotein (AFP) and CA19–9. There was also no association between receptor expression of the tumor cells and Child-Pugh score or patient survival, and there was also no influence of patient age or gender.

**Fig. 5:**
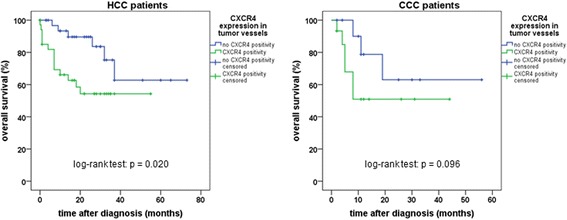
Overall survival of HCC or CCC patients with either no CXCR4 expression (*n* = 36 [HCC] or 12 [CCC]) or with CXCR4 positive staining (*n* = 35 [HCC] or 15 [CCC]) of tumor capillaries

A different picture emerged regarding SSTR5 and CXCR4 expression in tumor capillaries. HCCs displaying SSTR5-positive microvessels showed significantly higher Ki-67 values (*p* = 0.031) and more advanced grading (*p* = 0.023) compared to samples displaying no vascular SSTR5 expression. HCC patients with CXCR4-positive tumor capillaries exhibited significantly higher serum AFP values (*p* = 0.035) as well as a distinctly higher Ki-67 index (*p* < 0.001) than those with CXCR4-negative tumor microvessels. Moreover, overall survival of patients with HCC was significantly lower when CXCR4-positive tumor capillaries were present (log-rank test: *p* = 0.020; Fig. 5). Also in CCC patients, CXCR4 positivity of tumor vessels was associated with an elevated Ki-67 index of the tumor (*p* = 0.037). Also here, patient overall survival was reduced when tumor vessels stained positive for CXCR4, although (probably due to the relatively small number of cases) statistical significance was not reached (log-rank test: *p* = 0.096; Fig. 5). Between the SSTR5 or CXCR4 positivity of tumor microvessels and all other pathological or clinical parameters recorded no further significant association was found.

### Somatostatin and CXCR4 chemokine receptor expression in human hepatoblastoma and hepatoma cell lines

**Fig. 6:**
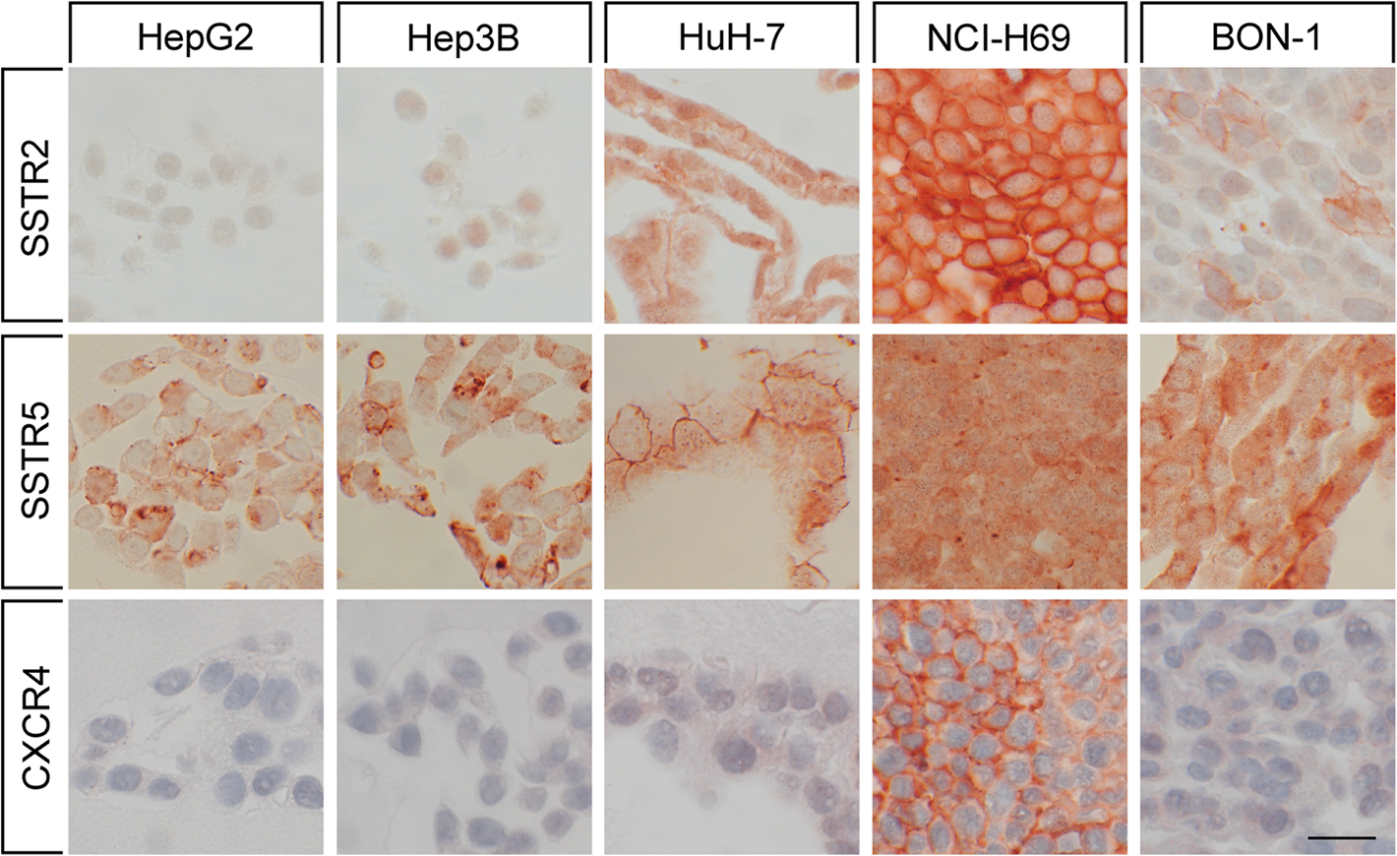
Somatostatin receptor 2 (SSTR2), somatostatin receptor 5 (SSTR5) and CXCR4 expression in the hepatoblastoma cell line HepG2 and in the hepatoma cell lines Hep3B and HuH-7 in comparison to the small cell lung cancer cell line NCI-H69 and the neuroendocrine tumor cell line BON-1. Immunohistochemistry (red-brown color), counterstaining with hematoxylin; scale bar: 20 μm. Representative photomicrographs of three independent batches are shown

Expression profiles of the SSTRs were quite divergent between the three hepatoblastoma and hepatoma cell lines investigated (Fig. 6). HepG2 cells displayed very weak expression of SSTR2 and moderate levels of SSTR5. Hep3B cells showed very weak expression of SSTR3 in addition to SSTR2 and SSTR5 staining. In contrast, HuH-7 cells stained positive for SSTR1, SSTR2, and SSTR5, but not SSTR3. The small cell lung cancer cell line NCI-H69 was devoid of SSTR1 expression but strongly expressed SSTR2, SSTR3, and SSTR5 (Fig. 6). BON-1 cells displayed weak SSTR1, moderate SSTR2, very weak SSTR3, and strong SSTR5 staining (Fig. 6). In all cases, both membrane-bound and cytoplasmic staining was observed.

With respect to CXCR4, Hep3B and HuH-7 cells displayed very weak expression, whereas moderate or strong staining was observed in BON-1 and NCI-H69 cells, respectively. Also here, both membrane-bound and cytoplasmic CXCR4 staining was evident. In HepG2 cells, in contrast, no noticeable CXCR4 expression was detected (Fig. 6).

## Discussion

### Patient characteristics

There was a strong prevalence of male gender (ratio of male:female patients = 4:1) in our study population, which corresponds well with literature data on HCC and CCC [9, 59, 60]. Also, median age of the patients at diagnosis (65 years) was in good agreement with the literature [9, 60, 61]. The median survival of patients with HCC in our study (21 months) was somewhat higher than that described in epidemiological studies [3, 4]. This difference may be due to the fact that we used tumor samples from patients who had undergone liver resection or liver transplantation, which usually implies a low tumor stage. Accordingly, the majority of our patients with HCC had stage I or stage II disease. In contrast, median survival of the patients with CCC was only half of that described in the literature [9], even though the patients in our study were subjected to surgical treatment.

### Somatostatin and CXCR4 chemokine receptor expression

In contrast to the existing literature, we detected SSTRs only infrequently in our cohort of patients. Previous studies had reported expression of all SSTRs – with the exception of SSTR4 − in 40–75% of HCCs, although sometimes with great variability and quite divergent receptor expression profiles [12–17]. Because at least moderately strong receptor expression intensity (i.e. an IRS ≥6) is mandatory for clinical utility, our results indicate that very few patients with HCC (about 10% of patients) are suited for SSTR-based diagnostics or therapy. This is in good agreement with the disappointing results of the majority of the trials to treat HCC using somatostatin analogs (see e.g. [29–36]). One reason for the discrepancies between the immunohistochemical data in literature and our staining results may be that different antibodies have been used. Whereas in the present study well-characterized monoclonal antibodies were employed, the great majority of immunohistochemical investigations in literature have been performed with polyclonal antibodies from different commercial and non-commercial sources (which may also explain the great variability in the SSTR expression profiles described). We rated the staining by means of the IRS, taking both frequency and intensity of expression into account, and only samples displaying an IRS value ≥3 were considered positive. Other studies provided no information to indicate which method was adopted to measure staining frequency and/or intensity. On the other hand, there may also be differences in tumor SSTR expression depending on the ethnicity of the patients and/or the etiology of the disease. The few trials reporting beneficial effects of somatostatin analogs in patients with HCC were either from Greece or Asia and comprised a high percentage of hepatitis B or C cases [16, 25–28]. This observation is mirrored by a meta-analysis [35] showing that octreotide has been ineffective in trials performed in Western countries but has significantly increased survival in Chinese studies with a high proportion of hepatitis B or C cases. Correspondingly, in an American study [34] no overall benefit of octreotide LAR in patients with HCC was observed, although one subgroup of patients showed significantly better survival than other subgroups. Some of the patients in that study were of Asian descent and had a history of hepatitis B infection. Our study included only Caucasians and only two of the 71 HCC patients had a history of hepatitis B infection. However, in both of those patients, no SSTR or CXCR4 expression was present. Because the issue still remains to be resolved, further studies using well-characterized monoclonal antibodies in a much larger set of patients to compare different ethnicities, HCC etiologies, and histopathological subgroups are necessary in order to identify patient subgroups that may benefit from somatostatin analog therapy. In our HCC patient cohort with mainly alcohol-related liver disease, SSTRs were not expressed at substantial frequencies or levels. Thus, because of the high costs of lifelong injections of long-acting pan-somatostatin-analogs [62, 63], such treatments should not be regarded as a standard therapy in such patients.

Our study is the first to analyze CCCs for the prevalence of the different SSTR subtypes. Thirty percent of the CCC samples displayed moderately strong SSTR expression (SSTR1 in most cases). Hence and in contrast to HCC, CCC may be well suited for SSTR-based treatment. In such instances, however, pan-somatostatin analogs should be preferred.

In contrast to the generally low levels of SSTRs, CXCR4 was more prevalent in HCC samples, although few tumors (6%) displayed moderately strong receptor expression. Similar to SSTRs, CXCR4 staining was more common in CCC than in HCC, and 11% of the samples displayed moderately strong CXCR4 expression. Again, there may also be differences in CXCR4 expression in HCC depending on the ethnicity of the patients and/or the etiology of the disease. In contrast, apart from a few positive Kupffer cells, normal liver tissues were largely devoid of CXCR4 expression, which is in agreement with the literature reporting higher CXCR4 expression in tumors compared with surrounding “normal” tissues [38–44].

The direct comparison of the receptor expression profiles between HCC and CCC (representing two different primary liver tumor entities with different etiologies) revealed a significantly higher SSTR1, SSTR2, SSTR5 and CXCR4 expression in CCC as compared to HCC. The most striking difference was seen with respect to SSTR1 expression. Whereas in HCC SSTR1 was expressed only in a very few cases, 67% of the CCC showed an IRS value ≥3 points. Thus, SSTR1 could serve as an additional marker to distinguish HCC from CCC and to identify the CCC portion in mixed tumors.

Neo-angiogenesis is an important factor in tumor growth, progression, and metastasis. Thus, targeting of angiogenic pathways has proven to have antitumor effects in a variety of cancers [64]. Because HCCs are highly vascularized tumors, they represent good candidates for antiangiogenic therapy. In HCC, however, the multi-tyrosine kinase inhibitor sorafenib displays only moderate effects in most patients and is not considered curative [65]. Furthermore, almost all clinical studies with other multi-tyrosine kinase inhibitors have failed [66]. Therefore, alternative antiangiogenic substances are currently under investigation for use in HCC treatment. In CCC, no respective treatment option currently exists. We found that CXCR4 was intensely expressed on tumor vessels in about 50% of both HCCs and CCCs. In HCC, CXCR4 staining of the tumor vessels was significantly associated with poor patient outcomes. A similar tendency was seen also in CCC. The presence of CXCR4 on endothelial cells in HCC has been observed previously [39, 47], although there was no association with tumor stage or grade, and specific targeting of the CXCL12/CXCR4 axis as an antiangiogenic strategy in HCC was recently suggested [67]. For CCC, similar observations have not been made so far. Although CXCR4 was not substantially expressed by HCC or CCC tumor cells, the distinct presence of CXCR4 on tumor capillaries in many cases may open up new possibilities to indirectly target such tumors by depriving them of their supportive environment.

### Somatostatin and CXCR4 chemokine receptor expression in human hepatoblastoma and hepatoma cell lines

In literature, SSTR expression in human hepatoblastoma or hepatoma cell lines has most often been evaluated in HepG2 cells. Previous studies observed expression of SSTR2 [13, 18, 68, 69], SSTR5 [13, 68], and SSTR3 [13, 18, 68] in HepG2 cells. Hep3B cells were not previously investigated with respect to SSTR expression. In HuH-7 cells (as in our experiments) previous studies reported expression of SSTR1, SSTR2, and SSTR5, but not that of SSTR3 [13]. Overall, our data on SSTR expression in these hepatoblastoma/hepatoma cell lines fit well with expression patterns obtained from HCC tumor staining. Similar to the results for the HCC samples, the most prominent SSTR in the hepatoblastoma/hepatoma cell lines was SSTR5, and (as in the tumors) expression of SSTRs in hepatoblastoma/hepatoma cell lines was weak compared with the expression levels observed in NCI-H69 and BON-1 cells.

With respect to CXCR4 expression, our results confirm previous findings showing CXCR4 expression in Hep3B and HuH-7 cells [43, 44, 70–72]. Regarding HepG2 cells, data in literature are controversial. While some authors were able to demonstrate CXCR4 expression [70–72], others were not [44]. According to our findings, all three hepatoblastoma/hepatoma cell lines only weakly expressed CXCR4 compared to NCI-H69 cells, which, again, fits well with our HCC staining results.

## Conclusions

In our study population, CCC, but not HCC, may be suited for both SSTR- and CXCR4-based treatment attempts. Because of the predominant expression of SSTR1, pan-somatostatin analogs should be preferred. In both HCC and CCC, indirect targeting of the tumors via the CXCR4 expressed on the tumor capillaries may represent an additional promising therapeutic strategy.

## Additional file

Additional file 1: Figure S1. Positive controls for immunostainings. Typical examples for positive control immunostainings for somatostatin receptors (SSTR) and CXCR4. SSTR1, SSTR2, SSTR3 and SSTR5: pancreatic islets; SSTR4: human cortex; CXCR4: germinal center of a lymph node. Immunohistochemistry (red-brown color), counterstaining with hematoxylin; scale bar: 20 μm. (TIFF 16836 kb)

## Abbreviations

CCC: Cholangiocellular carcinoma
HCC: Hepatocellular carcinoma
SSTR: Somatostatin receptor
