# Supplementary figures and images for: Somatostatin and CXCR4 chemokine receptor expression in hepatocellular and cholangiocellular carcinomas: tumor capillaries as promising targets
Source: BMC Cancer. 2017 Dec 28;17:896. doi: 10.1186/s12885-017-3911-3 (PMC5745780; doi:10.1186/s12885-017-3911-3)

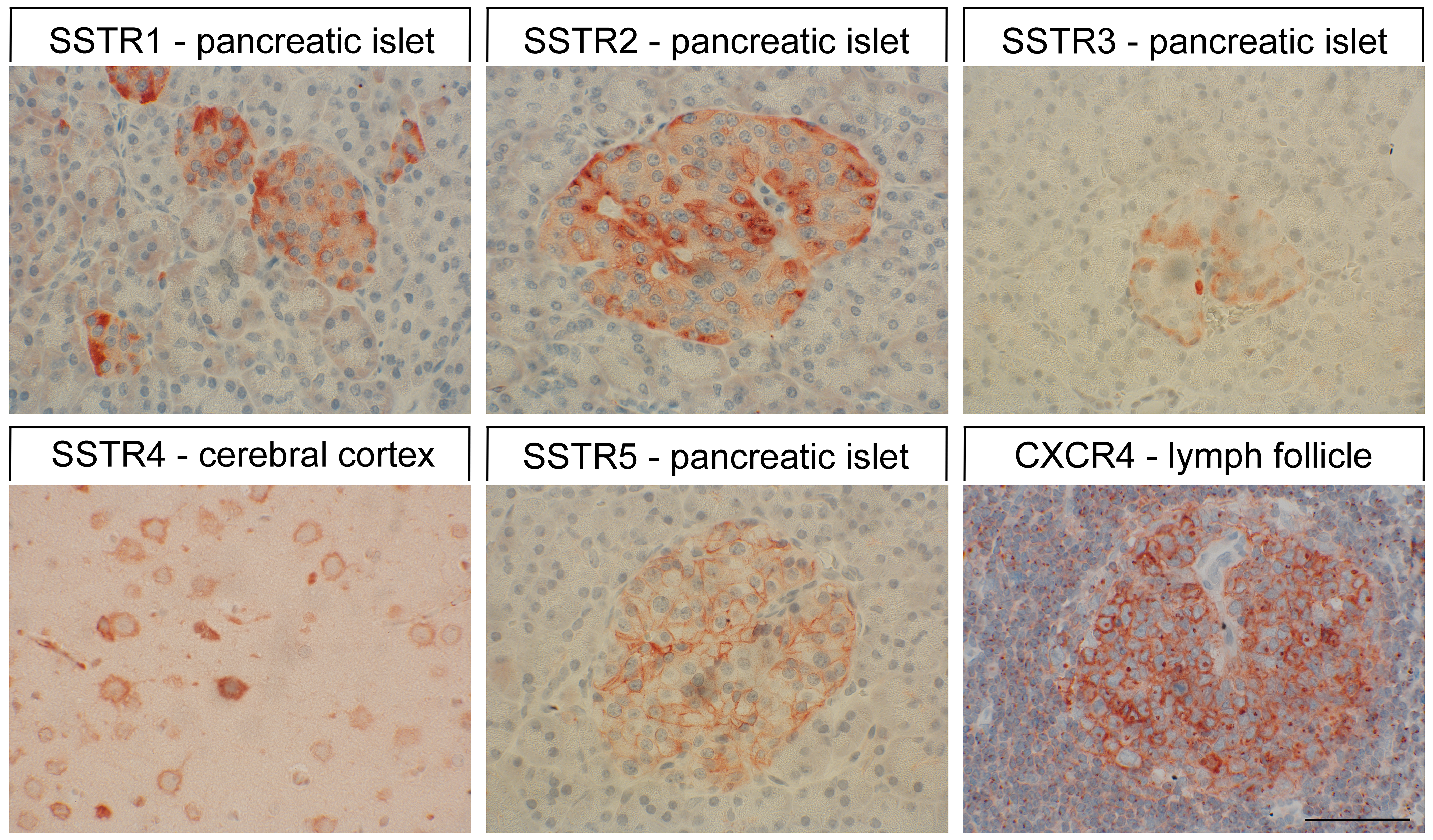

Supplement: Additional file 1: Figure S1. — Positive controls for immunostainings. Typical examples for positive control immunostainings for somatostatin receptors (SSTR) and CXCR4. SSTR1, SSTR2, SSTR3 and SSTR5: pancreatic islets; SSTR4: human cortex; CXCR4: germinal center of a lymph node. Immunohistochemistry (red-brown color), counterstaining with hematoxylin; scale bar: 20 μm. (TIFF 16836 kb) [file 12885_2017_3911_MOESM1_ESM.tif]
